# Child-centred care in practice: strengthening the right to play in public hospitals in Chile

**DOI:** 10.3389/frhs.2026.1824874

**Published:** 2026-06-22

**Authors:** Aleksandra Glos, Alejandra Santana López, Lilian Sanhueza Díaz, Ivonne Vargas Celis, Gabriela Piña Ahumada, Paulina Ramos Vergara

**Affiliations:** 1 Centre for Bioethics, Faculty of Medicine/Faculty of Law, Pontificia Universidad Católica de Chile, Santiago, Chile; 2 Facultad de Ciencias Sociales, Jurídicas y Humanidades, Universidad Gabriela Mistral, Santiago, Chile; 3 Facultad de Ciencias Sociales y Humanidades, Universidad Católica de Temuco, Temuco, Chile; 4 Escuela de Enfermería, Facultad de Medicina, Pontificia Universidad Católica de Chile, Santiago, Chile; 5 Escuela de Educación, Universidad Mayor, Santiago, Chile; 6 Centre for Bioethics, Faculty of Medicine, Pontificia Universidad Católica de Chile, Santiago, Chile

**Keywords:** children's right to play, hospital play, health policy, pediatric hospitalization, child-centered care, Chile

## Abstract

**Background:**

The right to play is a vital child's right protected by the UN Convention on the Rights of the Child. In hospital settings, play is essential to supporting children's recovery. Nonetheless, access to play is frequently uneven in hospitals. This research examined the availability of play opportunities in Chilean public hospitals, focusing on the factors that affect their accessibility, as well as healthcare professionals’ perspectives and attitudes toward children's play.

**Methods:**

This exploratory qualitative study collected data through semi-structured interviews with hospital professionals, supported by a self-assessment questionnaire, and through non-participant observations of hospital play areas. The data were analyzed using thematic analysis.

**Results:**

Healthcare professionals consider play to be a crucial element of child-centered care. Healthcare professionals understand that play significantly affects children's hospital experiences and strive to incorporate it as much as possible into their practice. However, the provision of this right in public facilities was inconsistent and influenced by various normative, institutional, cultural, professional, and child-specific factors outlined in this study.

**Conclusions:**

The growing recognition of play's importance among healthcare workers, combined with hospital management's efforts to promote play for children, indicates that Chile's next step should be to establish a formal minimum standard for play in pediatric health policies. This would require hospitals that serve children to provide such play opportunities. Drawing on hospital staff experiences, this study highlights the main factors affecting access to play in hospitals and identifies key elements for future policy development. Establishing this play standard could motivate further grassroots improvements in pediatric facilities throughout Chile and other Latin American countries.

## 1 Introduction

Play is a vital element of pediatric healthcare. It assists young patients in adjusting to new surroundings and re-establishes a sense of normalcy amidst the vulnerability caused by illness and hospital stays, which in turn enhances their resilience to stress (1–12). Play also supports children in healthcare environments by helping them understand medical information and assimilate it without fear (8, 10). Given the developmental and health benefits of play, it supports ongoing growth in children during hospitalization.

Previous research shows that healthcare professionals increasingly recognize the value of play in hospital settings. They understand play as a means to support children's emotional well-being and to reduce anxiety and distress during medical procedures, thereby contributing to more holistic and humanized pediatric care (4, 13, 14). Healthcare professionals also acknowledge that play can help children regain a sense of control and autonomy in environments often shaped by adult-driven routines. In this way, play is seen as central to protecting children's rights and promoting child-centered pediatric care (14, 15). An international Delphi study involving healthcare professionals from pediatric departments and hospitals across Australia, Canada, the USA, and eight European countries (16) further emphasized that healthcare providers consider play an essential clinical tool for communication and building trusting relationships with pediatric patients. The authors also found broad consensus across healthcare professions in different countries regarding the value of play in pediatric care. At the same time, some studies caution that play has traditionally been marginalized within hospital care, which can limit its integration into everyday pediatric practice (14, 17).

### 1.1 Children's play in a human rights framework

Play is not only a child's essential need but also their right, as stated in Article 31 of the UN Convention on the Rights of the Child (105). From a legal perspective, the child's right to play stands out “for being uniquely recognized for persons below 18” [cf (18): 3]. This distinctive status highlights its position as a specific good of childhood (19), and as an important determinant of its overall quality. Indeed, the UN Committee on the Rights of the Child in its commentary 17 to article 31 [ (20) § 14, p. 6] emphasized the intrinsic value of play for dignity and “pleasure of childhood,” without however neglecting the instrumental benefits for children's health, wellbeing and “physical, social, cognitive, emotional, and spiritual development.”

In healthcare, the UN Committee on the Rights of the Child notes that Article 31 is directly linked to Article 24, which guarantees every child's right to the highest attainable standard of health. The Committee states that allowing children to exercise their rights under Article 31 during illness or hospitalization “will play an important role in facilitating their recovery” [ (20), §25, p. 9].

### 1.2 International standards for hospital play

Play is one of the basic standards for respecting children's rights in hospitals in developed countries. As outlined by the WHO Regional Office for Europe (21), this standard should include a well-equipped playroom, staff dedicated to supporting children through play (e.g., play specialists), and a range of play opportunities, including clowning, music, art, and pet therapy. It is also important for health professionals to incorporate instrumental play activities into therapeutic care services. Article 7 of the European Association for Children in Hospital (22) emphasizes that “children shall have full opportunity for play, recreation, and education suited to their age and condition and shall be in an environment designed, furnished, staffed, and equipped to meet their needs.”

Many pediatric hospitals in high-income countries meet standards (7–10, 23–25), with the UK leading since the 1950s when the Platt Report recommended visible playrooms on children's wards (26). Past studies recommended that all children's wards include a playroom visible from the hospital beds [ (27), p. 51]. This report led to infrastructure reforms and the establishment of children's play specialists, who became part of hospital staff in the 1970s (28). Other high-income countries, such as the United States, Canada, Australia, Ireland, Japan, and Denmark, have also made significant progress in promoting children's right to play in hospitals (8, 24). For instance, in 1960, the American Academy of Pediatrics recommended that pediatric wards include playrooms with toys, games, and books (29). Similarly, Canada's Pediatric Society (30) recommended employing child life specialists to address the psychosocial needs of hospitalized children (31).

### 1.3 Children's right to play in Chilean legislation and pediatric health policy

In Chile, similar norms, standards, and studies are still being developed. The country ratified the UN Convention on the Rights of the Child in 1990 and has made notable progress in recent years. A key development was the creation of the Children's Ombudsman in 2018 (Law 21067) (102), followed by the 2020 Law on the Guarantees and Comprehensive Protection of Children and Adolescents (Law 21430) (103). This law serves as Chile's domestic implementation of the UN Convention, emphasizing children as rights-holders rather than mere “objects of protection.” It also affirms play as a fundamental right (Article 44) and mandates access to public play spaces (Article 46). However, Chile's recent report to the Committee on the Rights of the Child (32), which highlights various efforts to promote children's enjoyment under UNCRC Article 31, does not mention any specific initiatives aimed at implementing the right to play within healthcare settings [cf (32)., 141; 159–162].

In hospital contexts, Chilean public health policy [ (33), p. 86] emphasizes the importance of children's right to play. It recommends that hospitalized children have the opportunity to play, recreate, and learn in ways suited to their age. Nonetheless, the policy leaves the implementation of this right to each hospital's discretion, noting that its realization depends on the “capacities of each hospital” (p. 86). Additionally, a key limitation of this policy is its focus solely on children aged 0–9, thereby overlooking the rights and needs of older children and adolescents to participate in age-appropriate play.

A review of English- and Spanish-language literature found no studies or reports on the implementation of children's right to play in Chilean hospitals across academic, governmental, or organizational contexts. Additionally, a recent scoping review of play interventions in pediatric hospitals (6) found that among 297 articles, 78% originated from high-income countries [see also (34)]. These results indicate that play, especially in vulnerable situations such as illness and hospitalization, remains an under-researched right for children in developing countries like Chile.

This study aimed to examine the implementation of children's right to play in Chilean public hospitals by collecting qualitative data on institutional arrangements and opportunities for children's play. Specifically, the study sought to: (a) explore the conditions and opportunities for play available to children and adolescents in Chilean public hospitals; (b) examine healthcare professionals' experiences and perceptions regarding children's play in these settings; and (c) identify the factors that facilitate and/or limit opportunities for children's play in Chilean hospital contexts.

## 2 Materials and methods

### 2.1 Study design

This study adopted an exploratory, qualitative approach (35) to investigate play opportunities for hospitalized children and adolescents in Chilean public health institutions, focusing on the factors that facilitate or hinder their access, as well as hospital professionals' experiences and attitudes toward children's play. This approach was suitable for deepening understanding of how the right to play is perceived and implemented in public healthcare. These are areas that have not been extensively studied in Chile and Latin America.

### 2.2 Setting

Convenience sampling with purposive orientation was employed at two levels: selecting hospitals and choosing participants.

For hospital selection, the sample included one public pediatric hospital – one of only four nationwide, all located in Santiago—and three public hospitals with pediatric wards (out of 69 nationwide) from three different regions. The goal was to encompass diverse regional, socioeconomic, and cultural backgrounds (see Table 1). We have covered three macro-regions of Chile—central, northern, and southern—operationalized through three administrative regions: the Metropolitan Region (central), the Araucanía Region (south), and the Arica and Parinacota Region (north). This selection was designed to capture Chile's geographic and socio-cultural heterogeneity by including: (1) a highly urbanized, densely populated central setting with high migrant concentration (Metropolitan Region), where two hospitals serving populations from different socioeconomic contexts were purposively selected: one in a high-income municipality (low poverty rates) and one in a socioeconomically vulnerable area (substantially higher income and multidimensional poverty); (2) a southern region (Araucanía) with a high proportion of indigenous Mapuche communities and higher structural poverty levels, and (3) a northern border region (Arica and Parinacota) characterised by migration flows and a strong presence of indigenous Aymara populations. Together, this sampling strategy ensures variation across urban–rural divide, poverty levels, indigenous representation, and migrant presence, thereby supporting the study's aim to reflect diverse socioeconomic and cultural contexts within Chile's public pediatric healthcare system.

**Table 1:** Characteristics of participating Chilean public hospitals.

Hospital	Type of hospital	Characteristics	Presence of a playroom
*Hospital 1* *Central area*	Pediatric hospital	High complexity, Child-friendly hospital	No
*Hospital 2* *Central area*	General hospital with pediatric services	High complexity	No
*Hospital 3* *Southern area*	General hospital with pediatric services	High complexity	Yes (in the psychiatric ward)
*Hospital 4* *Northern area*	General hospital with pediatric services	High complexity	No

In terms of participant selection, the study included hospital professionals from pediatric wards or units within general hospitals. These included individuals involved in decision-making on play space allocation and management, those responsible for developing technical or administrative guidelines on the rights of hospitalized children and adolescents, and professionals actively promoting children's wellbeing and play through initiatives to enhance their quality of life. A total of 34 participants from diverse professions and hospital settings (see Table 2).

**Table 2:** Characteristics of participants.

Characteristic	Number (total, *n* = 34)
Sex of participants	
Female	24
Male	10
Profession	
Physicians	10
Nurses	8
Clinical psychologists	4
Occupational therapists	1
Physiotherapist	1
Actors	4
Teachers	4
Journalists	1
Role	
Clinical	18
Clown/Smile therapist^a^	5
Health educator	4
Hospital school director	1
Hospital director	1
Head of service	5
Foundation director	1

a In Chile, the term “smile therapist” (*sonrisólogo/a*) is locally used to refer to practitioners closely associated with hospital clowning who use humor, play, and emotional accompaniment to humanize healthcare. The term is often preferred over “clown” because it emphasizes well-being and care rather than theatrical performance. Some of our participants described themselves as “smile therapists” to reflect a broader commitment to the humanization of healthcare, extending beyond clowning alone to include other related initiatives and practices [Cf. Chile Crece Contigo (100)].

### 2.3 Data collection

#### 2.3.1 Semi-structured interviews

Semi-structured interviews with hospital professionals were conducted using interview guides developed through a literature review and aligned with the study goals. This method enabled conversations that were both guided and flexible, allowing exploration of participants' professional experiences and perspectives (36). While ensuring consistency across different regions, hospitals, and interviewers, the guide remained adaptable to include aspects that participants and interviewers found most meaningful. A total of 34 interviews took place from September 2024 to September 2025. Each session lasted between 30 min and 1 h 20 min, was conducted either in person or remotely, was recorded with permission, and was fully transcribed for analysis. All interviews were conducted by research team members.

The sample size was determined based on the richness and adequacy of the data in addressing the research aims. Data collection continued until the dataset provided sufficient depth and diversity to support meaningful thematic analysis of healthcare professionals' perspectives.

#### 2.3.2 Non-participant observation

The study also involved non-participant observations of 18 hospital playrooms included in the sample. Following space ethnography guidelines, these observations examined space and objects “from a perspective of data assemblage, where other data sources are investigated that can give insights into a broader picture of life and human activity” [(37): 621]. Including this method, consistent with the qualitative approach, enabled researchers to document equipment, location, accessibility, and overall space suitability for various types of play, providing valuable data for comparison with interview findings. Trained research assistants conducted observations in all play areas of the selected hospitals between September 2024 and September 2025 using a matrix aligned with research objectives. This matrix included information about the accessibility of the playroom, infrastructure and layout, equipment, spatial design, usability, and the characteristics of the children's play in each space. The areas observed included gardens, playgrounds, hallways, and waiting rooms with toys and bookshelves, and hospital schools. Each session lasted one hour and was documented with photographs (showing the space without children) and field notes. The handwritten field notes offered detailed descriptions of the space and its usage (38). Using a matrix aligned with research objectives, 18 play areas across four hospitals were observed.

#### 2.3.3 Self-assessment questionnaire

The self-assessment guide served as a complementary descriptive tool within the qualitative design. It was developed in accordance with the WHO (21) guidelines, “Children's Rights in Hospitals: Rapid Assessment Checklists,” which the research team translated into Spanish. The checklist operationalizes children's rights standards in hospital settings in line with the United Nations Convention on the Rights of the Child and was used to provide a standardized framework for documenting institutional practices related to children's access to play. After each interview, participants received a guide to completing their self-reports. Rather than serving as a standalone quantitative instrument, its use enabled greater consistency across hospital sites and supported a more comprehensive understanding of the findings by bringing together insights from interviews, observations, and institutional self-assessments, while remaining aligned with the study's qualitative and child-rights-based approach.

### 2.4 Data analysis

In the initial phase, we familiarized ourselves with the data by repeatedly reading the transcripts and taking analytic notes, highlights, and comments. During the second phase, the first author conducted the initial coding cycle, marking text segments with codes, organizing them into a matrix to facilitate mapping codes to themes, and refining the codes. This process followed Saldaña's (39) first-cycle coding, encouraging active engagement with the data. Both deductive and inductive approaches were used, with a focus on semantic and latent meanings. Using this framework, coauthors identified patterns through iterative second-cycle coding, with each coder focusing on data from a single hospital for in-depth analysis. In the third and fourth phases, themes and subthemes were developed by reviewing, merging, mapping, and renaming codes, creating thematic matrices for each hospital. These themes were collaboratively refined to ensure alignment with the research questions and consistency across cases. The final phase involved reporting the findings as a coherent analytical narrative that combined interpretive commentary with illustrative data extracts.

The observational data were analyzed using a simplified version of this method. Initially, all field notes were reviewed, followed by the identification of key themes. A new grid was then created to record the most significant findings and to supplement the interview data. This second grid gathered the most critical observations to identify patterns and differences between hospitals and to compare them with interview insights. The data from observations and interviews were analyzed and discussed collectively during team meetings.

To analyze the self-assessment questionnaires based on WHO guidelines, the data were entered into an Excel spreadsheet, integrating descriptive and categorical self-reported data. For each item, the percentage of positive responses per hospital was calculated, considering “TRUE” as a positive response and summarizing response patterns through frequencies and percentages. This process enabled comparison of compliance levels across hospitals for each item, a strategy commonly used in descriptive analyses of public health data (40, 41).

The quality of the analysis was maintained through team discussions, cross-reading and review of analyses, commenting, and keeping reflexive notes. To ensure trustworthiness, we used Braun and Clarke's 15-point checklist for good thematic analysis (42, 43), which is provided in Supplementary S1.

The findings were further interpreted in light of Lott's (44) STAR framework, which supports the implementation of children's right to play. This globally recognized implementation guideline provided a useful lens for situating local hospital experiences within broader children's rights and policy discussions. It enabled a more systematic analysis of the normative, institutional, and practical factors that could impact future policy and practice enhancements in Chile.

### 2.5 Ethical safeguards

This project received approval from the UC Health Sciences Scientific Ethics Committee, along with a written resolution certificate dated 18th April 2024 (Ref 231218019). It also gained approval from the Ethics Committees of the respective hospitals. All participants provided written informed consent, and their voluntary participation was fully respected. Interview data were transcribed under confidentiality agreements. Data from non-participatory observations were coded and used exclusively for research purposes, with confidentiality maintained at all stages.

A more detailed description of the study methods is provided in the published study protocol (45).

## 3 Results

Our results revealed a deep understanding of the importance of play in pediatric care and a longstanding commitment to providing children with optimal opportunities for play. Participants of the study cited several institutional initiatives that indicate growing recognition of the value of play among hospital management. Despite these efforts, opportunities for play, particularly free play—unstructured and not driven by educational, medical, or other adult-imposed goals—remained limited across all sources and institutions studied. To guide future public policies on hospital play, we identified factors that either hinder or promote the availability of play opportunities in public hospitals. These findings are organized into three main themes: (1) Play as the core of child-centered care; (2) The current state of play in Chilean hospitals; and (3) Elements affecting children's right to play.

### 3.1 Play is the foundation of child-centered care

Child-centered care is an approach in healthcare that focuses on children's individual needs, preferences, and perspectives (46). Unlike traditional paternalistic models, it promotes respect for children's rights and recognizes them as active participants in their healthcare choices (47). Additionally, child-centered care extends beyond a purely biomedical focus to encompass physical health and emotional, social, and developmental well-being (48). This study's findings show that acknowledging children's right to play helps ensure healthcare practices align with the principles of child-centered care.

#### 3.1.1 Play allows children to remain children

A recurring theme in our study was that play allows children to be seen as individuals rather than merely as “*numbers*” or “*medical diagnoses*.” As one respondent noted:

“It is crucial—crucial because, through play, children can truly stay children. At the hospital, they are treated as patients: children in beds, in units, with specific diagnoses, illnesses, and procedures. Do you understand? That is how they are viewed here, but play helps them remain children. They are no longer just ill beings, or defined by diagnoses, or undergoing treatment.” (Hospital 1, Interviewee 3, latter abbreviated as H1 I3)

Healthcare professionals emphasized that supporting a child's right to play is especially important in the highly regulated hospital setting, where most activities are mandatory (as one participant put it, “*the needle is mandatory, the medicine is mandatory, every two hours they come to take their temperature, their blood pressure—everything is mandatory”).* (H1, I7).

In this highly regulated environment, the healthcare team “began to realize that if a child is going to experience prolonged hospitalization, they are still a child. Playing is also their right, and therefore, we cannot deprive them of it”. (H4, I1).

#### 3.1.2 Play encourages a child-friendly quality of care

Interviewed professionals often reiterated that play places the child at the center of medical practice:

“[Play] is about making the hospital a friendly place. (..) I believe we have a duty to consider what is important to children, not from our perspective of what we think is important, but from their perspective (..). If we want to be a friendly hospital, we must include the element of play.” (H2, I3)

Play also contributes to the child-friendly quality of care by fostering personal relationships with children. As a respondent put it, play “*shows [children] that we are persons who care for them as persons* and helps to *connect with them on a personal level*.” (H1, I1)

This personalized, child-centered approach makes medical care more human and engaging:

“Through play, the attention given to children is warmer, of higher quality, more humanized, it has to be more humanized (..), because with children you need to bond with them to connect, otherwise you are working under pressure, with crying, irritability, and all that.” (H2, I3)

#### 3.1.3 Play and child-centered logic

As our participants emphasized, play places the child at the center of medical care and enables health professionals to move beyond a purely biomedical and paternalistic model of healthcare—one that focuses predominantly on physical aspects of illness and positions “*the doctor at the top of the hierarchy and the patient at the bottom*. *The importance of play lies in moving beyond a paternalistic, adult-centric logic.”* (H2, I3)

Many participants in our study highlighted the role of play in empowering children in medical care.

“Playing helps substantially with autonomy and problem-solving. Play is essential because it allows children to express their feelings” (H1, I2)

Participants in our research characterized play as an essential way to share children's medical information in a manner that is appropriate and engaging for children:

“Many procedures can be performed through play. Even something as minimal as giving a medication can be done differently so that it looks like a game and the child feels understood—feels like a child.” (H2, I3)

Play-based information sharing not only empowers young patients but also positively impacts their mental health and overall well-being:

“Through play, children often overcome their fears and push beyond their limits. When I show a child through play that something will be better for them, or when they are able to do it themselves, it makes a difference. (..) During a dressing change, they can see what I am doing or participate by showing where it hurts, how it hurts, or what could be done to prevent it. Many times, children relax—much more than they otherwise would.” (H2, I3)

#### 3.1.4 Play is a foundation of holistic pediatric care

Many professionals in the study viewed play as a core element of holistic care, encompassing not only biomedical but also mental, social, and cognitive dimensions.

“Play involves mental, social, physical, and educational health, all of which are encompassed within the concept of the humanization of healthcare.” (H2, I2)

#### 3.1.5 Play and physical health

Play has a powerful, direct, and long-term impact on children's physical health: “*When you play, you move—well, depending on the game—but usually you do. You run, for example. Imagine how beneficial that is for children who are overweight or obese*.” (H1, I6)

For this reason, the same professional later suggested that play should be treated as a health service in the hospital: I “*would install football fields and basketball courts—that is, I would include them almost as a form of healthcare provision*.” (H1, I6)

In response to the potential objection that the movement-related benefits of play are irrelevant in limited hospital spaces, where the most vulnerable children are often confined to bed, the following story should be recalled:

“There was a baby, about two or three years old, in the ICU. My colleague and I entered the room singing a very soft song. The child then sat up, and while the doctors were gathered around, the child began to dance and move. All the doctors were completely surprised and said, “Where did he get that strength from?” Because this was a child who appeared to be terminally ill.” (H2, I2)

#### 3.1.6 Play and mental health

Our participants emphasize that play is important for the mental health of pediatric patients, especially since illness and medical interventions are stressful for children and adolescents:

“…play is important for mental health; a child who is hospitalized is truly subjected to a great deal of stress because it is something unknown, with unfamiliar people… If we have an opportunity to have fun despite all the negative aspects (…) we can contribute a small grain of sand to make this experience more pleasant because they are children.” (H4, I1)

Consequently, many health professionals, particularly in clinical psychology and psychiatry, supported the incorporation of play into hospital mental health treatment services.

“[Play] should be included also as a mental health policy, now that mental health is such a prominent topic and has been given so much importance. Play is fundamental to mental health; it is essential.” (H1, I7)

#### 3.1.7 Play and the social dimension of health

Play enables children to connect, share, and build friendships. It also supports their social and moral development. Our respondents noted that play fosters inclusive communities that are fun and *free from bullying*. One participant shared how play promotes respectful interaction and mutual care among ill and hospitalized children:

“When children have a patch, a visible suture, or bandages—as is often the case in the burn unit—play helps lift them up and raises their self-esteem. When we are playing, every child is simply another child here. Among themselves, they don't see illness or bandages, and in that sense, they are extremely generous. If there is a child with a deformity or without hair, to them, it is just another child. What they see is the boy or the girl behind it.” (H1, I4)

Play serves purposes beyond fostering inclusion; it also imparts lessons on rules and values. For many children, play is their first experience of understanding fairness and the importance of adhering to rules (49). As noted in our study:

“If we play a collective game, no one would cheat or do anything like that. For them, play is a serious matter. There are children—especially those in short-term care—who may have broken many social rules, but when it comes to the rules of the game, they do not negotiate.” (H1, I4)

#### 3.1.8 Play and cognitive development

Play is a natural and enjoyable way for children to learn. Through playful exploration, children can experiment with their environment, adapt to new situations, and learn in engaging and effective ways (49).

“Everything—everything is achieved through play. All of a child's learning, all of their cognitive functions, are developed through play.” (H1, I5)

Our participants emphasized that play-based learning is particularly important in hospital environments, since long hospital stays can restrict children's chances for cognitive development: “*I feel that children have to learn through play; that's why, in terms of the work I do at my hospital, I try to make sure that this is not lost*.” (H4, I1)

### 3.2 State of play in Chilean hospitals

This overview of public hospitals across various regions of Chile highlights differing socioeconomic, urban, and cultural profiles. It is complemented by Supplementary S2, which presents findings from non-participant observations of play areas across four institutions, and Supplementary S3, which presents results from the self-assessment questionnaire based on the WHO Rapid Checklist (21), completed by participants afterwards.

#### 3.2.1 Hospital 1: leading pediatric hospital in a privileged zone

Hospital 1 was the only facility whose institutional policies formally recognized the right to play. One contributing factor was its being the only specialized pediatric hospital in our study, which made it naturally more attuned to children's rights, their specific needs, and overall well-being: “*I think an important factor is that it is a pediatric hospital, so you have to take it as a given that play is important*.” (H1, E6)

An additional key factor was that the hospital 1 implemented the Child-Friendly Healthcare Initiative model, created by UNICEF and the Royal College of Pediatrics and Child Health, which aligns healthcare practices with the UN Convention on the Rights of the Child (50).

“The right to play and children's rights are enshrined in our strategic planning. We have a hospital that works with a rights-based approach. We have developed courses and training programs so that staff are aware of at least the basics of the Convention on the Rights of the Child and how we become guarantors by working in a pediatric institution such as this.” (H1, I3)

An additional facilitator was the hospital’s location in an economically privileged area of the Metropolitan region^1^.

“This pediatric hospital is located in the eastern part of the city, a well-off area where we benefit from many advantages, not only economic ones but also the support networks of a hospital that receives strong backing from people, foundations, and institutions. I think that, of course, we are not on the periphery; we are in a place that works in our favor.” (H1, I3)

However, despite formal recognition of the value of play, most participants considered the provisions of this right insufficient, “*particularly regarding children*’*s free play”:*

“I think we would like to see more done, such as giving children more space; I think we have a debt in that regard, in terms of allowing them to play freely, because there are more and more restrictions due to hospital infections and other technical issues, and all the talk about cost/benefit. Cost/benefit for whom? I feel that we have a debt in terms of free play.” (H1, I1)

The stories shared by health professionals also reflected a sense of loss stemming from the disappearance of play spaces after the COVID-19 pandemic. This includes a hospital library with an integrated toy library and a welcoming surgical ward that was vital in calming children before surgery. One of the professionals indicated:

“That was a very intense and powerful place. It allowed not only children but also parents to reduce their anxiety, and play helped reconnect the child with who they used to be. Through this language that is so unique to them—play—they were able to disconnect from everything that was happening: uncertainty, insecurity, and fear. In that situation, play was a lifeline. And we lost that too.” (H1, I3)

Although there was no dedicated playroom, the hospital featured a well-stocked school with games, books, and handmade materials suitable for all ages, based on field observations. Moreover, the hospital 1 offered numerous recreational initiatives—far more than those in other non-pediatric hospitals included in our study. As one respondent noted: We have, for example, “*circuses and theater. We brought ballet, cinema, and MIM, to name a few”.* (H1, I3) Hospital clowns and volunteers also made an invaluable contribution to the playful atmosphere in the hospital:

“Now, we don't have a library, but we have volunteers who encourage play areas, read stories to the children, invite them to do different crafts, and the children ask when the ladies are coming. The storytellers are women who come with little baskets that make a noise. So, the sound of the basket is the sound of play coming.” (H1, I2)

All nine observed spaces in hospital 1 were accessible to wheelchair users and children with reduced mobility, with wide aisles and doors, and well-equipped with ramps. However, there were no accommodations for children with visual impairments.

#### 3.2.2 Hospital 2. clown-led play spaces in a general hospital in a vulnerable zone

Opportunities for play were considerably more limited in the general hospital, situated in one of the most socially vulnerable areas of the Metropolitan Region, where multidimensional poverty rates are particularly high^2^ (51). In Chile, socioeconomic disparities are spatially distributed across urban areas, with more advantaged zones separated from more disadvantaged ones, reflecting social and cultural segregation.

Resource scarcity significantly limits children's opportunities to play:

“I think it's a matter of resources, because awareness is there, but the problem is that there are no resources. In fact, sometimes, we don't even have the [medical] supplies we need to do things. So, it's really a matter of resources. Some organizations and groups bring items—for example, with Easter approaching, they’ve brought small bunnies all this week to decorate the hallways or cardboard eggs. There are organizations that bring things and help quite a bit, but it's still not enough.” (H2, I7)

In resource-scarce conditions, opportunities for play largely rely on hospital staff's initiative. At hospital 2, two smile therapists are especially notable for their ongoing dedication and inventive use of limited resources. One of their most remarkable projects involved converting a former hospital dumping site into a play park for children and their families:

“When we arrived in 2008, (…) this park was a dump inside the hospital (…). A few years later, we decided to seek help from the hospital community and the municipality to restore green spaces. So, the dump was cleared, the space was reclaimed, and it was cleaned (…)”. (H2, I6)

This initiative was enabled by collaboration among the municipality, hospital management, and individual contributors. As one of the key initiators recalled: “*The mayor gave us a beautiful house with a bathroom, and that's where we started making the little paths, planting together with the mothers, working with the families*.” (H2, I2)

The area transformed into a lively play park bordered by native trees, featuring diverse play equipment, children's artwork, and memorials honoring deceased children. This creates a symbolic space for gathering, fostering tranquility, enjoyment, and emotional support. Observations indicated that, despite minimal equipment, the space serves its purpose effectively (e.g., toys and playground equipment), that there are places to sit in the shade of the trees, and that the temperature is very pleasant in summer. These characteristics made it a suitable place for guided and free play. This space facilitates various forms of play, such as “*looking for bugs under stones, swinging, chasing one another, playing hide-and-seek, and role-playing, including feeding dolls, administering injections, putting them to bed, and caring for them.*” (H2, I6) However, the space is difficult to access for people with reduced mobility due to the uneven terrain, narrow paths, and its distance from the hospital's main building. Additionally, the nearest toilet is accessed through steps. A wheelchair user could get to the park with help, but could not make full use of the space.

#### 3.2.3 Hospital 3: high-complexity general hospital in the poorest region of Chile

Hospital 3, located in a region of Araucanía that for decades been constantly identified as one of the poorest in the country, particularly with respect to multidimensional poverty^3^, and has reported similar challenges:

“We recently received an email stating that the contracts had expired due to financial reasons. The idea is to resume the [laughter therapy] program at some point in the future, when finances allow. (..) That's the proof that the thread was cut at its weakest point, so one of the first things to go was the clowns.” (H3, I1)

Another factor restricting play at the hospital 3 was that it was a general, high-complexity facility serving the entire region:

“This hospital is a regional, high-complexity hospital, and its focus is on the adult population. Therefore, things are done with the adult population in mind, and, by extension, there have to be general protocols and rules that apply to children (…). So, if someone asks for a toy, it becomes a second- or third-level priority, or even lower. Because the institution is not oriented in that direction. I would love there to be a pediatric hospital.” (H3, I10)

**Figure 1:**
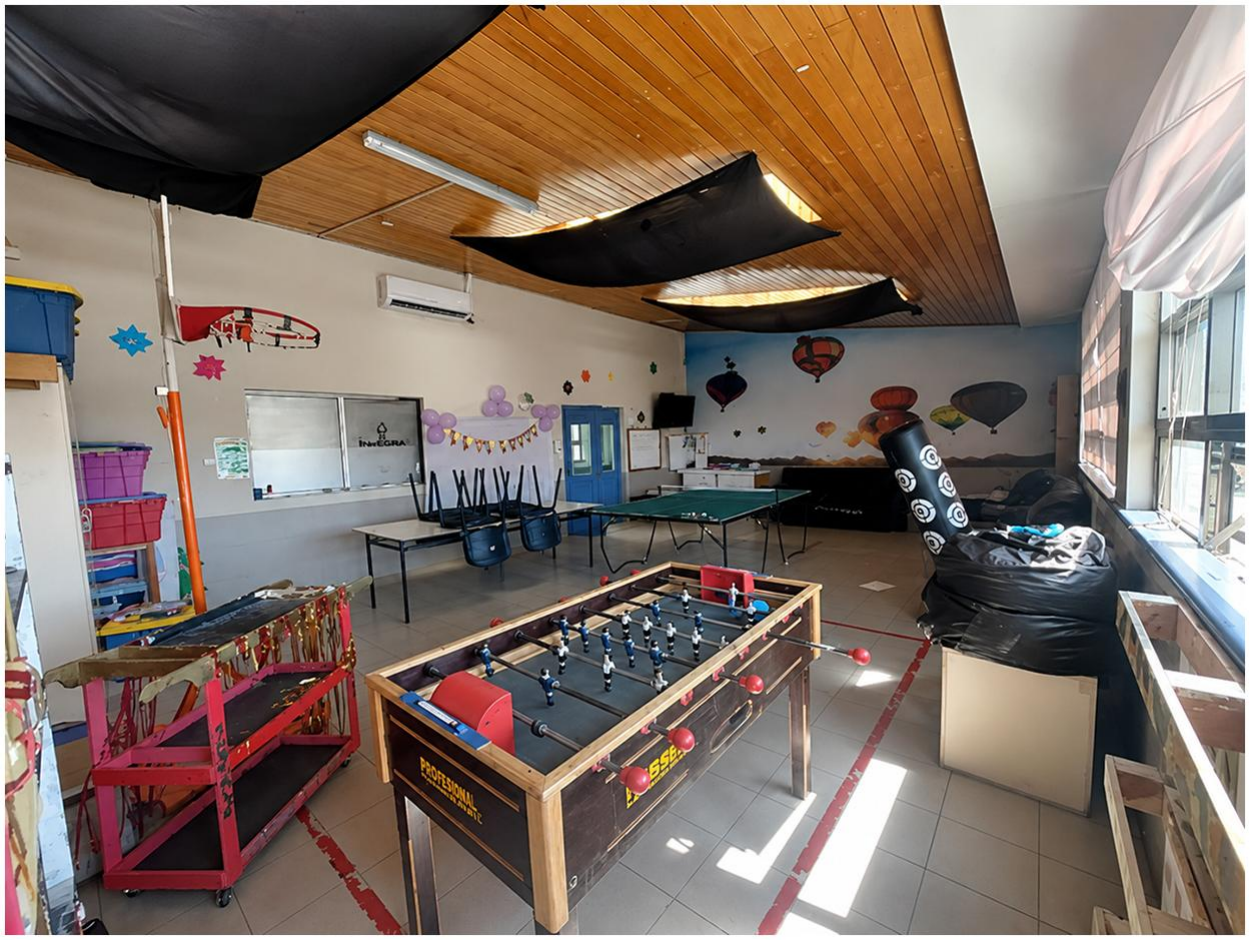
Mental health and psychiatry service-run playroom for children and adolescents. Alt-text: Well-lit playroom equipped with a foosball table, ping-pong table, and mural of aerostatic balloons.

Despite limited institutional policies and ongoing resource challenges, dedicated health professionals, supported by foundations, volunteers, and public funds, launched several child-friendly initiatives. Their efforts made the hospital 3 the only one in our study with a dedicated playroom within the Child and Adolescent Mental Health and Psychiatry Inpatient Service (Figure 1). This was significant because most play areas were designed for early childhood, with few or no facilities for adolescents. Field observations showed the presence of video games, musical instruments, and a ping-pong table—amenities likely to appeal to older children and teenagers. The space is accessible for children with reduced mobility with wide doors and ramps, though, as in the other hospitals, there are no indications for children with visual impairments.

The medical team consistently emphasized the importance of play for everyone involved in child care, challenging the notion that play is beneficial only for children. As one staff member highlighted:

“No, at the institutional level, within the hospital, free play is not promoted. I am very conclusive about that; it is obvious. However, within our service, we do try to promote it as much as possible”. (H3, I12)

#### 3.2.4 Hospital 4: the role of the hospital classroom in a high-complexity general hospital

Hospital 4 is in Arica, in Chile's northernmost region, which borders Peru and Bolivia and experiences significant humanitarian needs.^4^ This is a general, high-complexity hospital where the pediatric department represents a small portion, reflecting limited pediatric services and the lack of a dedicated playroom as a priority space

“It's not a very large service either. In the outpatient area, there's a corridor; it's not like there's a nice space or an internal courtyard.” (H4, I2)

Although there is no dedicated playroom, hospital 4 has a classroom, which was described as a gift to the hospital because it did not previously exist in Arica.

“Last year, the hospital classroom arrived, and we divided the ages according to the work to be done. They took children from 5 to 14 years old because Chile Crece Contigo covers up to 4 years, 11 months, and 29 days, which we handle”. (H4, I1)

This classroom observation highlights various play stations equipped with materials such as books, art supplies, and musical instruments, providing children with a wide range of play opportunities. Space supports their overall development as they engage in diverse activities:

“Yes, when the hospital classroom staff are present, depending on the patient, they go to the units and deliver materials, games, or activities in the child's ward. If the patient's clinical condition allows, they come to this room and share”. (H4, I3)

Access to this room, which serves as both a classroom and a stimulation room depending on the time of day and staff availability, is restricted. The room is locked with a key held by the nursing unit and is available only during the school term (March – December) and on weekdays when the supervising professional is present. Staff shortages also limit play activities opportunities:

“We only have about 15 min a day to carry out these activities with each student, and the children are left wanting more. You tell them, “No, we have to stop now—we’ll see each other again tomorrow.” But they say, “Miss, we’re having fun.” So, in a way, their energy lifts for a moment, and then they go back to feeling down again”. (H4, I4)

During school holidays and weekends, children staying in the hospital lack access to classroom activities and have only limited options for games or drawing materials. Many hours when the room is closed and unused occur because there's no staff available to supervise children*. “They can't take books out; (..) they can't come here to play because they’d be alone”.* (H4, I3)

The room is accessible for children with reduced mobility, with doors wide enough for wheelchairs and stretchers. However, during the observations, the professionals commented on the lack of toys for children with paralysis.

Beyond the hospital classroom, a mobile play cart sponsored by Chile Crece Contigo visits children in their rooms to facilitate play activities. Nevertheless, the availability of program materials affects this service. Professionals note how much children enjoy these activities:

“They like it and wait for the “cart lady: “Where are the toys?” They show their drawings and crafts proudly—it does them a lot of good”. (H4, I4)

### 3.3 Factors determining the right to play in Chilean public hospitals

To guide future hospital play policy, we identified five factors influencing play in Chilean healthcare facilities: (1) normative, (2), institutional, (3) cultural, (4) professional, and (5) child-related (see Table 3).

**Table 3:** Factors that determine play opportunities.

Category	Factors determining play
Normative factors	Public policy and financing for play Public programs supporting play Hospital school programs
Institutional factors	Play spaces Playful design Play equipment Financing Human resources Play policy Hospital rules Safety protocols
Cultural factors	Institutional culture Recognizing the value of play Adult-centered culture Cost-effectiveness Biomedical model of care
Professional factors	Profession Role Time Personality Training
Child-related factors	Age Health condition Dual function of technology

#### 3.3.1 Normative factors

Professionals from various hospitals emphasized that the lack of a public policy on play is a key factor limiting children's opportunities to engage in and enjoy this activity: “*My criticism is more of a macro level, referring to the ministerial and state institutions. (…) It [the policy of play] does not come from any higher institution (…)”.* (H1, I3)

Participants' criticism reflects the ambiguous status of the right to play in Chilean law and health policy. Although both the Chilean children's rights law (Law 21.430) and national pediatric policy (33) formally recognize the importance of play, these principles are not consistently implemented across institutions responsible for children's well-being, including hospital settings. This partial recognition of play at the legal and policy levels reveals a systemic undervaluation of play (52, 53). In hospital settings, it reflects entrenched distinctions between forms of care considered medically necessary and those viewed as supplementary or non-essential. It also points to a broader conceptual separation in which play is treated as an isolated children's right, rather than as fundamentally interconnected with other rights, including the rights to health, participation, education, and, more broadly, children's dignity.

This normative marginalization at the public level is further reflected and reproduced in institutional decision-making across healthcare settings, shaping how hospital managers understand and respond to children's needs in pediatric care. As a result, play is often treated as an optional or expendable addition to treatment rather than as an integral component of pediatric care. As one healthcare facility director candidly admitted, if play were compulsory, space would be allocated for it despite all obstacles:

“From a more practical standpoint, I think we could be required to have a play space of specific dimensions, located either inside or outside the hospital. A playroom, a library, or a recreational space—whatever the name may be—is something that could simply be established as a requirement.” (H1, I10)

The underrecognition of play at the normative level has particularly severe consequences for underfunded public healthcare systems. In the absence of a clear public policy, the financial and organizational responsibility for providing opportunities for play is shifted onto individual institutions and their personnel: “*I think implementation of play depends on public health policy, because that's where the necessary resources can also be assigned*.” (H3, I1)

In Chile, socioeconomic inequality is structured both along the public–private divide and spatially across different areas of the country (54–57). Within this context, hospitals in under-resourced areas often face significant challenges in sustaining dedicated play initiatives and infrastructure, particularly during periods of financial strain, institutional restructuring, or crisis. Participants described how systemic pressures, including pandemic restrictions and budget limitations, frequently resulted in the reduction or suspension of play opportunities for hospitalized children, such as the closure of playrooms or the discontinuation of clown programs. In such contexts, resource scarcity further reinforces the prioritization of biomedical interventions over psychosocial forms of care. These dynamics were reported, in different forms, across all the institutions examined. Reflecting on these challenges, one professional noted:

“(…) public policy is important. I think funding to have hospital clowns is extremely important (…) it is an urgent matter (…) people ask us, “and the clowns, and the clowns?” and “what about the clowns and the smile therapists?” and today we do not have funding for the clowns; we had funding for 20 years, and from one day to the next we were left without it (…)” (H2, I2)

**Figure 2:**
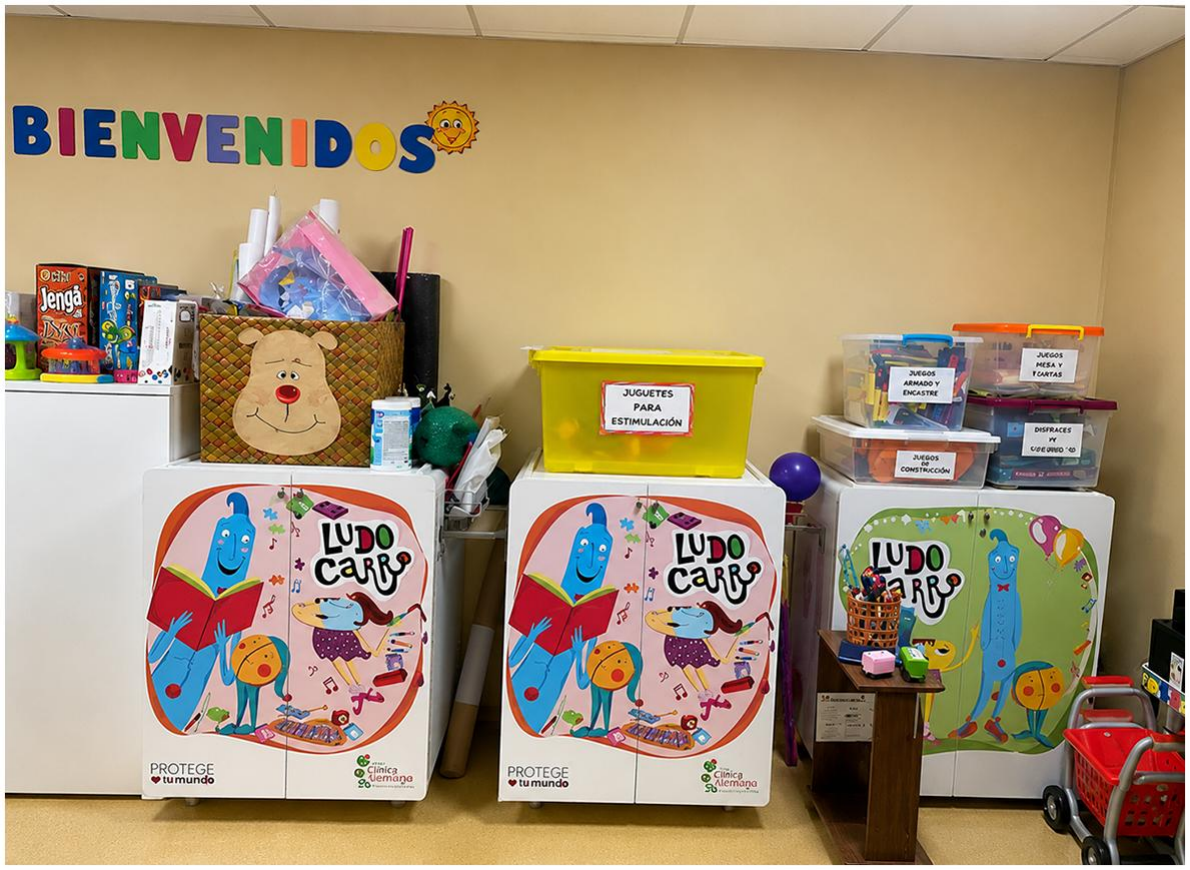
Mobile play cart (“*ludocarro*”) funded by the Chile crece contigo program. Alt text: Three play-carts with colorful decor and plastic boxes with toys and art supplies sitting on top.

Despite reported limitations in Chile's pediatric health policy, the country has launched additional initiatives that are praised for expanding opportunities to play. The most consistently praised program across hospitals was Chile Crece Contigo (Chile Grows with You), part of the Social Protection System, which is overseen by the Ministry of Social Development and administers, coordinates, supervises, and evaluates it (58). As reported, the program, whose guidelines include *“creating play areas in hospitals to lessen the impact of the healthcare process on children and their carers (..)”* (H3, E4) provides its own play-related materials (see Figure 2) and employs staff who care for the biopsychosocial needs of hospitalized children.

Chile Crece Contigo, one of the national cross-cutting programs that operate outside the hospital system, also provides materials to hospitals so that hospitalized children—even those who do not attend the hospital school—still have opportunities to play. They regularly visit the hospital, offering children books, toys, pencils, and modeling clay—items that help them express themselves and have fun. They also organize activities whenever staff are available to encourage them.

Although the program received very positive evaluations across hospitals, it also had drawbacks, including limited outreach. “*We hope to see it implemented at larger service levels or units”*. Another limitation was its focus on very young children (0–9 years old): **“***It is very focused first on the mother and birth, then on the perinatal period, and then onward. I feel that the program becomes thinner as the child grows older”.* (H1, I10)

**Figure 3:**
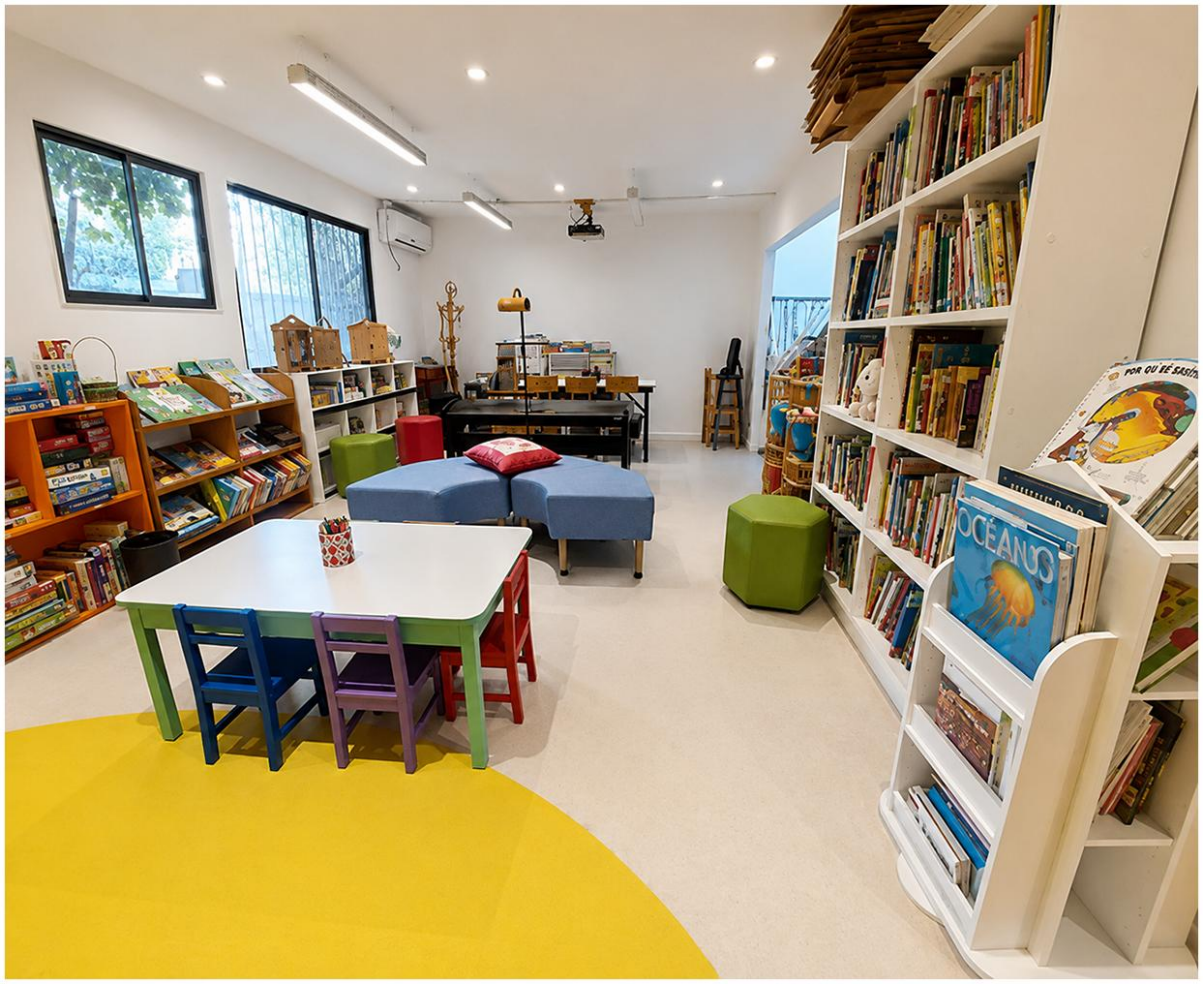
Child-friendly library of a hospital school. Alt text: Library equipped with a table, chairs, seats, and small shelves with books and board games.

Another public policy that improves children's access to play in hospitals is the General Education Law, which mandates the establishment of schools in medical settings (58). The significance of hospital schools in encouraging play is profound (Figure 3). The strongest evidence of this is the following:

“We have had children who came to school even to sleep. These were children in their final moments, in the last stage of their lives, in their final days, and they wanted to come to school anyway, in wheelchairs. They would stay in the classroom listening, sometimes falling asleep, talking and playing, resting—because they wanted to be with their classmates and teachers, to talk, and to share time together. It has been beautiful and incredible, and it has happened several times”. (H1, I5)

While hospital schools do offer play opportunities, they cannot replace a dedicated hospital playroom. This is due to their primary educational focus and several infrastructure challenges identified in our study, including being closed on weekends and summer holidays, and often located outside the main hospital building. Access is limited to specific children, those with medical approval and the ability to be accompanied, and typically only to children staying for extended hospital stays.

“It is also a closed space; it's not as simple as saying, “Let's go to the school playground.” It is more limited (…). This means that if, on a given day, all the beds are full and staff are extremely busy, no one will take the child down to school, and the child will feel that absence. There is still no policy that treats access to contact with other children or access to play materials as equally important as access to healthcare.” (H1, I6)

#### 3.3.2 Institutional factors

A common factor restricting the right to play in Chilean hospitals is the shortage of dedicated physical play spaces:

“So, I feel that another barrier is physical space. It happens to our hospital, and it also happens to other hospitals across Chile: they are overcrowded and lack physical space. Well, just yesterday, we were talking with the head nurse, and she obviously said, “I don't want to lose this [play] space.” (H4, I1)

At each hospital, play areas were characterized as fragile, contingent, and subject to administrative decisions, rather than as a right for children to play. These spaces were often difficult to locate due to inadequate signage or limited accessibility for children with mobility impairments. This highlights a structural gap in hospital design, planning, and policies for play spaces—a problem many participants would like to see addressed. Notably, suggestions for improved hospital design appeared to draw on foreign models.

“I wish we could demolish the hospital and start from scratch, so that even the space itself would be designed for play. This is already the model in some international hospitals, where even the stretchers are different, the walls are much more playful, and so are the waiting rooms. That would be wonderful. I mean, if we could dream that on each floor of the hospital there was a playroom, it would be wonderful.” (H1, I6)

**Figure 4:**
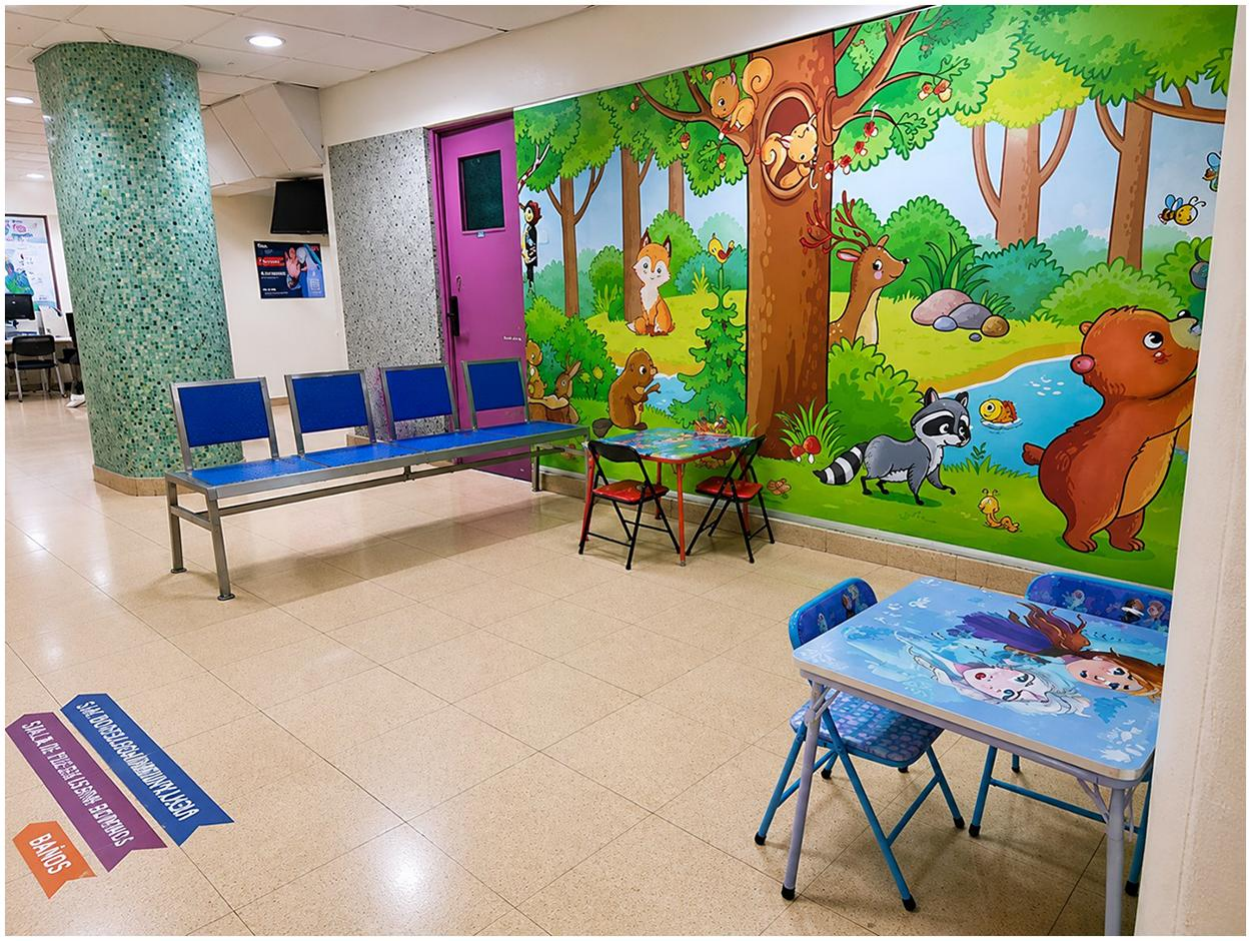
Children's waiting room, pediatric nephrology and peritoneal dialysis unit.

Many interviewed health professionals emphasized the significance of child-friendly decor throughout the children's wards. Colors, drawings, and other playful design features were used to make the hospital environment less intimidating and to demonstrate concern for young patients' comfort and well-being (see Figure 4).

“Having the walls a bit more cheerful because visually it makes a big difference when you arrive at a place and everything feels brighter.” (H3, I2)

Although all participating hospitals included some child-friendly decorations, these were generally perceived as limited, incomplete, and lacking in systematization, making them more aspirational than functional or effective.

“The waiting rooms, for example, for radiology, were improved a bit. The walls were painted. It's not a playroom as such, but at least there was an effort to make the waiting room much friendlier, so children are in a slightly better environment.” (H2, I1)

Many hospitals also noted that child-friendly initiatives could be extended to all services and that the green areas around the hospital could be utilized more effectively.

“There are green areas here, around the hospital (.) Perhaps one could (…) create a physical space for play, a space that is safe and has oxygen connections.” (H3, I9)

Most equipment is acquired via donations, volunteer efforts, external programs, or intra-hospital solidarity initiatives, rather than through systematic institutional processes.

“Fundraising campaigns are organized, and some staff members also purchase toys for the children with their own money.” (H3, I11)

Participants noted the absence of a dedicated budget for play, which hinders its implementation and signals that it is a lower priority within institutional goals. Despite ongoing funding challenges, many healthcare workers showed their commitment to the right to play by devising innovative solutions, such as reusing materials.

“(…) We have very few resources (…) [we need] a cost centre to obtain resources (…) not “Fisher Price” toys—we don't need that—but the minimum (..) We use many reusable resources, bottles as rattles, empty containers (…), we create a lot of material, but certain resources are still needed.” (H1, I2)

Another limited resource across hospitals is human capital. Participants indicated ongoing staff shortages, with health professionals often having to assume multiple roles and lacking specialized training in play-based practices.

“There isn't any staff who can dedicate themselves exclusively to play (…) sometimes someone can come and bring a game or something, but you can't stay with them all the time because of work demands.” (H4, I3)

As mentioned, only the pediatric hospital (hospital 1) had internal policies and initiatives that actively supported play. In contrast, professionals from general hospitals explicitly reported the absence of any formal play policy:

“(…) as an institution, I haven't seen any effort to implement play as a right (…) there's no institutional plan to generate opportunities for all patients as a right—there really isn't.” (H2, I5)

All hospitals reported numerous infection-control and biosafety measures that limit children's play opportunities, highlighting how play is systematically subordinated to clinical priorities.

“(…) it's restricted (…) there are certain protocols regarding care to avoid hospital-acquired infections (…) when we bring stimulation materials to the room, we have a whole protocol for disinfecting materials, and parents are always reminded that the materials brought to their child cannot be shared with another child in the room.” (H3, I4)

Many hospital professionals highlighted that the risk of infection is a significant limitation impacting play among hospitalized children. In the hospital environment, where sick patients and concerns about hospital-acquired infections and healthcare-associated infections are present, play is somewhat limited*.*

This fear of contagion was driven by the COVID-19 pandemic, and even as the global emergency has ended, it persists and manifests in new forms. “(…) *There are, as they call them, ‘bugs,’ new ones, so this fear of contagion has separated us a bit (…) people look at each other inside the hospital with more fear, even though masks can be used and all that* (…).” (H1, I4)

Another health-related factor limiting play is pathological severity: “*it is understood that the child has the right to play because they are a child, but it is not something we see as a right in the critical situation they are in (…) that is, in the ICU, they are all ventilated—impossible, there is no play. In the intermediate care unit, they sometimes come straight from the ICU and are still dealing with acute pathology, so play is not seen as something which I have to ensure.”* (H4, I4)

Simultaneously, many experts maintained that play, a core need and right of children, must not vanish in more complex clinical settings. Rather, it should evolve to align with the child's physical, emotional, and social abilities. They highlighted that adults are responsible for customizing play activities to suit the pediatric patient's health status.

As one effective strategy to reach even the most vulnerable children, the following method was mentioned: “*sometimes the intervention is through a glass (…) the glass can be wonderful because on the glass we can draw smiles, we can—there are beautiful interventions from behind the glass, and also interventions where, as I was saying, the last thing the child sees is the face of a clown when they go under anesthesia.”* (H1, I7)

Many health professionals emphasized that serious health conditions should not be seen as barriers to playing. Instead, play can offer emotional and physical benefits for children with significant health issues. An example of this therapeutic potential of play is illustrated by the following case:

“(…) we have seen it with several patients. For example, patients whom we need to start moving and who don't want to, but when you take them to stimulation therapy, that's when they do start to move. Or children who require neurological rehabilitation, for example, after traumatic brain injury with sequelae, who are in bed and don't want to do anything, spending all day on their phones, but when they are taken to the playroom, they start to move and play with balls, and that obviously makes their rehabilitation progress much faster.” (H4, I2)

#### 3.3.3 Cultural factors

Regarding institutional determinants of play, respondents mentioned not only infrastructural and organizational factors but also cultural elements, referring both to broader societal views on childhood and play and to the institutional cultures of particular hospitals in Chile:

“Working within an institutional culture that supports the right to play seems to me to be just as important as making sure that everyone involved in healthcare understands and incorporates the importance of play into healthcare practice.” (H1, I8)

A major obstacle to children's playtime was the failure to recognize its significance for the well-being of young patients, along with its marginalization as a child's right:

“I think we lack awareness that it is a right. Sometimes, the positive impact of play is unknown, not only for a child in a hospitalization context. We need to strengthen the idea that it is a valuable tool, a form of children's everyday expression through which they build their world, relate to others, and express their needs (…). It's left to each professional's discretion, especially in pediatric areas. We need to keep this in mind as something important and a right, so that, as staff, we might even think (…) are we violating that right?” (H3, I4)

A common theme among hospitals, especially among the most dedicated professionals who recognize the importance of play and actively promote it, was the ongoing challenge of gaining recognition for play's value from other professionals and hospital management more broadly. As one respondent noted, “*It's kind of my struggle in my day-to-day work”* (H1, I1)

This challenge often emerged in relation to colleagues or managers who questioned or minimized the relevance of play within the hospital setting: “*I think that the issue of play is still somewhat associated among professionals with a kind of waste of time (…).”* (H2, I6)

Many professionals attributed this lack of recognition of the importance of the right to play to broader cultural factors, such as a pervasive adult-centrist culture. As one respondent explained: “*We live in an adult-centrist society, and everything that belongs to children's own forms of behavior seems to lose value in adulthood (…) it is devalued (…), and those who attribute value to play are seen as ‘odd ones out.’”* (H3, I3)

Another cultural factor observed was the prevalence of cost-effectiveness models in healthcare, which overlook quality care considerations:

“I would start from the point of view that in healthcare, above all else, the goal is always to obtain the greatest benefit at the lowest cost. And play does not fit into that dynamic; in other words, play falls into a secondary dynamic.” (H1, I9)

Additionally, the medicalization of care models—focused mainly on treating physical illness while overlooking mental, social, cognitive, and environmental aspects of health, as outlined by the World Health Organization (1948)—was noted as a barrier to recognizing and implementing children's rights:

“I don't know if anyone really sees it as right, to be honest. I don't think so, because sometimes we have to go and ask, like, “hey, please, bring things for this child to play with.” So, it's not always just there. It often feels like care is more medicalized, I would say, rather than truly holistic—unless we, as a team, are actively concerned with making it so.” (H4, I2)

#### 3.3.4 Professional factors

In general, the health professionals interviewed recognize the importance of play for hospitalized children:

“Everyone on the teams has tried to (…) optimize children's rights, provide better care to patients, and encourage play. So, we ask them to bring their toys, music, tablets, stuffed animals, whatever makes them feel at home. (…) For example, they wake up in recovery, and their stuffed animal is next to them (…) with the same little patch they have, the same little cap they’re wearing, and they pick it up, hug it, and say, “We’ve already had the operation, Pepito.” (H1, I8)

While health professionals acknowledge the importance of play, not all participate in it themselves. Some believe that “others” are responsible for engaging in play. This relates to the roles assigned to each professional:

“For example, therapists and physiotherapists are responsible for motor rehabilitation, and within their activities, through play, they achieve those rehabilitations. But, for example, I, as a nurse who is responsible for administering medications and other procedures, for me playing is the last of my priorities (…) Now, if I can play while I’m doing the procedure, fine, but play is not going to be my first priority; for those other professionals, it is.” (H3, I7)

Some professionals view play primarily as a tool, using it for rehabilitation, to educate pediatric patients, or to distract children during painful procedures. While this instrumental approach underscores play's importance in delivering child-friendly, stress-free medical care, those who value play for its inherent benefits argue that such uses prioritize adult needs over children's wellbeing and dignity.

“They are doing a procedure and ask me to stay there and try to distract the child. I think things get mixed up; the purpose of play gets muddied.” (H3, I4)

Psychologists, educators, and clowns are among the healthcare specialists most dedicated to integrating play into their practice.

“The teachers from the hospital school (…) bring tools so children can be distracted, play with them, and we also take part a little—we’re more into stickers, drawings, and things like that.” (H3, I2)

Among medical professionals, many believe their role does not involve play. As one of them noted, “*in my direct role, [I play] probably not much, because it's mainly an assistive role; that is, the time one spends with the patient is focused on treating the pathology they have.”* (H1, I1)

These professionals are constrained by heavy workloads and not enough time to engage in play: “(…) *we focus on the respiratory aspect; the time between patients, given that many patients are allotted a maximum of 10 min. Between assessing and providing care, those 10 min are used up. But sometimes, one still makes the time to play a little*.” (H2, I7)

At the same time, many of the physicians and nurses interviewed exhibited a deep commitment to play, both as a practical tool and as an intrinsic value.

“Then, professionals such as doctors, nurses, and nursing technicians—I imagine it depends on each person; some are more playful and others less so. Each uses the tools they feel help them connect. I have seen, for example, the use of songs, stories (…) to do something like give an injection (…). But really, I think that depends very much on the personality of each professional.” (H2, I6)

To raise awareness of the significance of play among healthcare providers, many participants emphasized the need to integrate play into medical education. This should go beyond theoretical knowledge by fostering practical skills through hands-on experience.

“One of the things we’re trying to do as a specialty is to have pediatric trainees rotate through child and adolescent psychiatry for at least one month. So, if we manage to raise sensitivity – because I think the theory is already there (…) Play is important because it's an act that has importance in itself, as part of the developmental process, and all that. But you necessarily have to see it in practice; you have to play with a child, because otherwise it won't work.” (H3, I12)

#### 3.3.5 Child-related factors

Both interviews and ethnographic observations pointed to children's age as a key factor restricting access to play opportunities.

“We tend to think of a certain type of child – if we had to assign an age, we’d say around eight years old—and, of course, the older ones slip through the cracks, as do the babies. So, the play spaces we haven't really been designed for the full range of childhoods and adolescents that come to this institution.” (H1, I3)

Engaging adolescents in play is often more complex and challenging. It involves more than just providing toys; it requires connecting with their world, understanding their needs and concerns, and building trust through meaningful interactions. As one professional explains:

“Because through play, you open pathways to interact with children; with adolescents, it is more difficult because there isn't as much play—there you turn to other types of humor and other kinds of jokes with the kids.” (H4, I3)

With few exceptions, adolescents were mostly excluded from playful activities. Ethnographic observations revealed that the most common equipment found in playspaces was small tables with chairs, books, and toys for very young children. Although the park at hospital 2 can be enjoyed by people of all ages, the playground equipment is too small for adolescents. Two of the hospital schools observed, in hospitals 1 and 3, were notable exceptions. These spaces were equipped with books for older children and adolescents, arts supplies, and musical instruments that children of all ages can enjoy. However, in both hospitals, the materials are only available during school hours. Finally, the Child and Adolescent Mental Health and Psychiatry Inpatient Service at hospital 3 was also remarkably well-equipped with books, instruments, and art supplies for children of all ages, as well as video game consoles, a ping-pong table, and a football table (Figure 1).

“I think that adolescents in this hospital currently do not play. If there are games, they are for small children. (…) from the doctor's discourse, it's like “you’re already big, you have to take responsibility for your treatment,” as if you were already on the verge of being an adult (…) I don't agree that adolescents don't like to play (…) it has to do with the type of games that are offered.” (H1, I6)

In terms of accessibility, the observations revealed that all but one of the spaces observed (the play park in hospital 2) were easily accessible to children with limited mobility. Most places had wide doors and ramps when needed. In this regard, children are not excluded from play opportunities. However, regarding the equipment of the play rooms, more could be done to accommodate other kinds of disabilities, for instance, the stimulation room in hospital 4 lacks toys for children with paralysis. Also, the most commonly available material, books for young children, relies heavily on sight, so they would be less suitable for children with visual impairments. Therefore, disability is a limiting factor for opportunities for play, as not all play spaces offer suitable equipment for children with different disabilities.

A recurring theme across all hospitals and interviews was the ambivalent role of technology in children's play and overall well-being. One professional described it as “*a double-edged sword”* (H1, I7): it can benefit children when used responsibly, but it can also harm their development and social skills. In many situations, technologies like tablets and mobile phones can serve as valuable opportunities for play:

“When a child undergoes chemotherapy, they have to spend eight hours in the hospital. So (…) if there is a child who is connected, I also arrive with my phone, and from there you create something (…).” (H1, I7)

Technologies can facilitate interaction between children: “*when they’ve been together for several days and are more or less the same age, they talk, and since they have phones, they play online”* (H4, I3). They can also be a more attractive tool for play and communication with adolescents: “*personally, I like video games, so I start playing video games with a more adolescent boy (…), and they start to relax, you start to talk with them, and they begin to open up and communicate thing*s.” (H4, I10)

Some professionals criticize the use of technology, particularly regarding the relationship between younger hospitalized children and their caregivers:

“They bring computers or tablets from home, and the mother is not bothered. And sometimes, the child watching videos is very small. So, there you have to stimulate communication, interaction, and relationships, which is much more enriching than the tablet” (H4, I2).

## 4 Discussion

This study explored how play is perceived and implemented in pediatric hospital care. Using an interpretive qualitative approach, it examined the factors influencing play practices in underfunded public healthcare settings. This research provides the first empirically based account of play in Chilean pediatric hospitals. The results shed light on how play serves as both a care practice and a negotiated institutional process, offering insights that are locally relevant while also contributing to global discussions on child-centered care.

On a global scale, this study reinforces existing evidence that hospital play positively impacts children's health and well-being in pediatric care. Although play is acknowledged as part of quality healthcare (21, 59), the global health community must consider it essential to healthcare rather than just a secondary right (10). Our research highlights how play fosters child-friendly healthcare. As Tonkin [(60), p. 3] insightfully observed, “Play is holistic in nature and promotes each aspect of health, including physical, social, emotional, mental, environmental, and spiritual health.” In line with earlier studies, our findings indicate that play benefits children's physical health (6, 61, 62), mental health (3, 9, 63–66), psychosocial health (7, 24, 25, 67, 68), and cognitive development within hospital settings (3). Play also enables children to remain children (11) and, as such, is vital for upholding other pediatric patient rights in a child-friendly way. By centering children in medical practice, play helps make pediatric care more humane, personalized, and meaningful from a child's perspective.

Across Latin America, research on pediatric care has consistently described play as a means of sustaining children's development, reducing the stress of hospitalization, and humanizing relationships between children, families, and healthcare teams. In Mexico, Hernández-Arenas (69) argues that play supports children's social, emotional, and bodily development while reducing anxiety during medical treatment and making hospitals feel less exclusively centered on illness. In Venezuela, Serrada Fonseca (70) contends that integrating playful activities into the educational care of hospitalized children is essential to supporting continuity in child development. Brazilian scholarship has likewise developed a strong body of work around play—particularly therapeutic play (*brinquedo terapêutico*)—and the humanization of care. Sossela and Sager (71) identify play as a fundamental right closely tied to children's physical and emotional well-being and as a resource that helps mitigate the effects of hospitalization. In a similar vein, Ciuffo et al. (72), in a qualitative study conducted in a pediatric hospital in Rio de Janeiro, shows that nurses and nursing technicians regard toys as valuable tools for reducing fear, easing tension, and fostering bonds between children and healthcare professionals. Taken together, this literature suggests that play is not a secondary or merely recreational activity but a central dimension of childhood health, development, and citizenship, whose meaningful exercise depends on adequate institutional and material conditions.

Despite growing recognition of play's importance to the quality of pediatric care, significant barriers still hinder its equitable adoption worldwide. Even well-developed, child-friendly systems like in the UK are described as “play-deprived” in some aspects (73). Interestingly, these challenges resemble those in Chile, including COVID-19-related disruptions, staff shortages, and inconsistent service delivery. Additionally, in many countries, teenagers often remain overlooked in healthcare, with limited access to age-appropriate play (16, 74). Reports from Chile, where professionals struggle to gain institutional recognition for play, reflect this international pattern (75). The publication of the General Comment on Article 31 was itself driven by the longstanding “poor recognition” (CRC 2, p. 3) of play, along with chronic underfunding and weak legislative protections in public settings (34). These findings indicate that the right to play, often described as “forgotten” and “neglected” (76–79), remains secondary to other rights deemed more urgent (80), including within public healthcare systems worldwide. This indicates that the global health community remains in an early stage of integrating the connection between children's right to play (Article 31) and their right to health (Article 24). In light of these considerations, our interpretive analysis of the factors that influence, limit, and facilitate play in public healthcare environments provides valuable insights worldwide to better realize this right.

This study builds on existing research on the implementation of children's right to play in hospitals by offering empirically grounded insights from a Latin American context, an area underrepresented in current literature (6, 34). At the normative level, participants identified the absence of a specific public policy as a key obstacle. Without minimum standards, the financial and organizational burden falls on individual hospitals and staff, making play programs vulnerable to budget issues, health restrictions, and administrative changes. These findings align with reports from the Office of the Children's Rights Ombudsman in Chile (52, 53), which argue that the State must actively create conditions that guarantee children's play, rather than merely recognizing it formally. They also reflect broader social perceptions that frame play as wasted time despite its importance for children's overall well-being (52, 53). In this sense, the findings reveal not only normative gaps but also a cultural hierarchy of priorities in which treatment, efficiency, and clinical control tend to overshadow practices associated with childhood, well-being, autonomy, and self-expression.

At the institutional level, both the literature and the present findings point to persistent structural limitations affecting the implementation of play in hospitals. Several studies conducted in Latin America report a lack of training for play practices among healthcare teams, limited implementation materials, insufficient adapted spaces, and the absence of systematic protocols (81–83). These studies further suggest that healthcare systems rarely institutionalize or provide permanent funding for such programs (84). Similarly, our findings reveal that Chilean public hospitals often fail to provide secure environments for play, with children's access heavily shaped by adult-controlled decisions, shortages of physical spaces, the absence of dedicated funding, reliance on donations, and a lack of specialized staff. Barriers related to biosafety have also been documented, particularly during infectious outbreaks such as COVID-19, when preventive isolation measures interrupted recreational visits and restricted access to hospital playrooms (85). This pattern aligns with Brazilian research on therapeutic play (71, 72), in which healthcare teams acknowledge the benefits of play but face heavy workloads, staff shortages, and insufficient play materials. Similarly, Serrada Fonseca (70), in Venezuela, points to the need for employing staff specifically responsible for organizing and supervising play within hospitals. Overall, the similarities across Latin American countries suggest that play provision in hospitals in the region is often sustained by partial, creative, and informal solutions rather than by stable, long-term institutional frameworks.

### 4.1 Study limitations

The primary limitation of this study is the exclusion of children's direct perspectives. Data were collected through interviews with adults and observations of playroom spaces, supported by a self-assessment questionnaire, rather than from children's own experiences. Although this approach was suitable for an initial exploration phase and for building institutional collaborations to support future child-focused research, it remains an important limitation. Future studies should focus on capturing children's views and preferences regarding hospital play to inform more responsive, rights-based healthcare reforms.

Given the partial availability of inclusive infrastructure and equipment observed in the hospitals, future research should explore accessibility accommodations for children with disabilities in greater depth. Beyond infrastructural adaptations, the specific support needs of children with disabilities should be addressed by ensuring the availability of adequately trained personnel who can provide play-based interventions tailored to their needs and facilitate their inclusion in shared play activities with other children. Hospitals are environments in which temporary or permanent physical, sensory, cognitive, and emotional disabilities are particularly prevalent and may become more pronounced. Therefore, accessibility accommodations—both infrastructural and interpersonal—should constitute an important component of hospital policies and research agendas. Future participatory and interdisciplinary studies should focus specifically on children with various disabilities and examine the availability, effectiveness, and lived experiences of inclusive play opportunities in Chilean hospitals.

Moreover, comparative studies between public and private healthcare systems are needed to help further clarify how socioeconomic inequalities shape access to play opportunities in Chile. Longitudinal research could also explore the long-term emotional, developmental, and psychosocial effects of play-oriented interventions in pediatric healthcare settings. Future studies should additionally explore the different uses and needs of play across age groups, as well as among children in diverse healthcare contexts and conditions, such as general pediatric wards, intensive care units, chronically ill children, and those receiving palliative care.

## 5 Conclusions

We conclude with initial evidence-based recommendations for future public health reforms to enhance play opportunities in pediatric settings. To structure these recommendations, this study draws on Lott's (44) STAR framework—comprising Space, Time, Acceptance, and Rights-informed—as an interpretive tool for translating empirical findings into practical guidance. Our recommendations are grounded in evidence from triangulated interviews, field observations, and institutional self-assessment data (Table 4).

**Table 4:** Recommendations for Chilean hospitals based on STAR framework.

STAR Framework		
Dimension	Main findings	Key recommendations
Space	Most hospitals lack dedicated playrooms and equitable access to play materials.	Establish mandatory playrooms in all pediatric wards; ensure public funding for equitable implementation; integrate playful design into broader hospital spaces (corridors, waiting rooms, gardens); and provide inclusive, accessible play materials for children with diverse needs.
Time	Hospital routines, medical procedures, and institutional pressures often limit opportunities for spontaneous and self-directed play. Access also depends on adults available to facilitate play.	Protect dedicated time for play within hospital schedules; formally recognize and employ play specialists and hospital clowns; implement institutional play policies and protocols that integrate play into pediatric care.
Acceptance	Play is often treated as optional rather than as a fundamental right of childhood. Adult-centered biomedical models may marginalize children's developmental and emotional needs.	Promote child-centered and rights-based care approaches; increase professional training on the value of play.
Rights-informed	The right to play is interconnected with children's rights to health, participation, and non-discrimination. Certain groups (e.g., adolescents, children with disabilities) face greater barriers to play.	Use play-based approaches in treatment and communication; involve children in designing play services; ensure inclusive and accessible play opportunities for children with diverse physical, cognitive, and emotional needs.

### 5.1 Space

The fundamental aspect of ensuring children's right to play is providing appropriate spaces for it. According to Yu et al. [ (25), 6], “Play spaces in children's healthcare settings are typically viewed as safe, homelike spaces that provide children with a sense of agency and normalcy within the broader (and, arguably, colder or less child-friendly) institutional healthcare environment.” Our findings further underscore the restorative benefits of these play areas, suggesting colorful, child-friendly hospital schools and outdoor play spaces may help foster feelings of peace and comfort among children.

Providing inviting, child-friendly, and safe play spaces is more than just a logistical issue; as Lott (44) rightly points out, it is “an issue of spatial justice” (p. 7). Public spaces, even those intended for children, often prioritize institutional routines over children's rights and developmental needs. In this context, our research identifies space as a fundamental condition for the realization of children's right to play in hospital settings, one that remains insufficiently recognized and implemented in Chile, underscoring the need for clear and enforceable public policy. This policy should require dedicated playrooms in all hospital wards that admit children, not only in the four specialized pediatric hospitals located in the capital, but also in the 69 general hospitals with pediatric wards across the country. Despite infrastructural constraints, participants emphasized that suitable play spaces could still be created if they were legally mandated, as reflected in the testimony of one hospital director in our study. The feasibility of this approach is further illustrated by the example of hospital schools, which, following their legal mandate in 2009 (Law No. 20.370) (104), now operate in approximately 75% of Chilean public hospitals (86). However, given the chronic underfunding of the public healthcare system and persistent territorial inequalities, equitable implementation would require sustained financial support from the Ministry of Health, particularly for rural and socioeconomically disadvantaged hospitals. In other words, providing play opportunities should not be left to the capacities of individual hospitals, as under the present pediatric policy (33), but should be made mandatory and supported by public funding. These places should also ensure the accessibility of stimulating play materials, responding to the needs of children of different ages, genders, abilities, and health conditions.

Beyond establishing designated playrooms as a minimum standard, hospitals should also promote play through broader spatial transformations across the healthcare environment. As demonstrated by Yu et al. (24), cafeterias, lounges, atria, gardens, corridors, and other shared spaces can support opportunities for play and social interaction, particularly for children who cannot access designated playrooms due to disabilities or medical restrictions. Similarly, observations conducted in Hospital 2 showed that even spaces not originally intended for children (and initially as uninviting as a hospital storage or dumping area) could, through the commitment and creativity of dedicated hospital professionals, be transformed into more welcoming and playful environments. Through relatively low-cost interventions—such as the use of colors, murals, playful furniture, and child-friendly design elements—both indoor and outdoor hospital spaces can become more supportive, stimulating, and inviting for children and their families.

These broader spatial transformations also require the active involvement of hospital professionals. Beyond advocating and actively participating in creating designated playrooms, professionals can support the integration of playful and comforting elements into everyday hospital spaces, such as bedside areas, waiting rooms, corridors, and outdoor areas. This, in turn, calls for interdisciplinary collaboration among healthcare staff, hospital administrators, educators, and play specialists or clowns to ensure that hospital environments are responsive to children's emotional, developmental, and social needs.

### 5.2 Time

Ensuring sufficient time is crucial for protecting children's right to play in hospitals (87). First, children themselves must have protected time for play within daily hospital routines, including opportunities to engage in free and self-directed play between medical procedures and treatments. Healthcare professionals should therefore remain attentive to how rigid schedules, medical routines, and institutional time pressures may unintentionally restrict children's opportunities for spontaneous play and leisure. Recognizing play as an essential component of pediatric care requires creating temporal flexibility within hospital routines so that children are not positioned solely as patients undergoing treatment, but also as children with developmental, emotional, and social needs.

At the same time, children's access to play also depends on the availability of adults whose time is specifically dedicated to facilitating and supporting play activities. Although free time between medical procedures is important, our findings suggest that this alone is often insufficient in highly regulated hospital environments, where opportunities for play are shaped by institutional constraints. Due to safety and biosecurity regulations, access to play is frequently mediated by the availability of accompanying adults, healthcare staff, volunteers, hospital clowns, or other professionals responsible for supervising children. Consequently, another key requirement for the realization of children's right to play is the presence of dedicated adults whose primary role is to facilitate play and whose working time is institutionally protected for this purpose. This finding further highlights the importance of formally recognizing and employing play specialists, hospital clowns, and other trained professionals who support play and children's psychosocial well-being in pediatric healthcare settings (88).

In line with the WHO (21) guidelines for assessing children's rights standards in hospitals, institutions should adopt formal play policies that allocate time for play in pediatric wards. Existing evidence further suggests that implementing formal play protocols and quality-improvement initiatives can help integrate play into everyday pediatric care routines (89). Hospitals should therefore establish clear institutional policies that include dedicated play sessions within daily ward schedules, provide guidance on age-appropriate and inclusive play practices, and formally recognize the role of professionals responsible for facilitating play. Embedding play within formal institutional schedules could help protect play opportunities from being marginalized by competing medical and administrative demands and would reinforce the recognition of play as an integral dimension of pediatric healthcare rather than an optional recreational activity.

### 5.3 Acceptance

Third, children's right to play depends on whether it is recognized as a fundamental right of childhood rather than merely an activity requiring adult approval. According to Lott [ (44), p. 15 ff], “permission” makes adults gatekeepers of play, whereas “acceptance” recognizes children as rights-holders who do not require adult approval to exercise their rights. This distinction has been key to determining access to play in hospitals. Notably, the only hospital with a fully equipped playroom was located in the most socioeconomically advantaged region, suggesting that, in the ongoing debate between redistribution and recognition (90), the core issue is less about resource redistribution and more about the recognition of children's rights. When play is fully recognized as vital to childhood and linked to child-friendly care, especially in public policy, it often triggers the mobilization of financial and institutional resources.

Consequently, accepting play as a right demands a broader cultural and institutional shift away from adult-centered, biomedical models toward child-centered, rights-based care approaches. This change entails rethinking hospital policies, routines, and professional training to promote play across different times and locations and to accommodate children's health conditions, including those of children with serious illness. Promoting such a shift may be facilitated by professional training and sensitizing healthcare professionals, hospital administrators, and support staff to the value of hospital play. Workshops, interdisciplinary training programs, reflective practice sessions, and experiential learning activities may help professionals better understand the developmental, emotional, communicative, and therapeutic significance of play in pediatric healthcare.

### 5.4 Rights-informed

Human rights frameworks generally recognize that rights are interconnected and indivisible (91). In line with this perspective, the UN Committee on the Rights of the Child emphasizes that the right to play is closely linked to children's right to health, although this interconnection is not yet consistently reflected in healthcare policies or practices. Recognizing this relationship in pediatric healthcare settings invites healthcare professionals to harness play in diverse forms—including physical exercise, sensory stimulation, serious games, and other therapeutic play interventions—as part of children's treatment, recovery, and rehabilitation processes (6).

The interdependence of rights also indicates that the realization of other rights can help ensure that children effectively enjoy the right to play. In this context, Article 12 of the UNCRC, which affirms children's right to be heard in matters affecting them, is especially relevant. This has important implications for policymakers and healthcare institutions alike: when children's opinions inform the design of play areas, schedules, and activities, play services are better able to respond to their needs and lived experiences. This also underscores the importance of including children's perspectives in research on the understanding and implementation of the right to play. Moreover, as play can strengthen children's meaningful participation in medical conversations and decision-making processes (92, 93), healthcare professionals should be encouraged to adopt play-based approaches to explain medical procedures, support mutual understanding, reduce fear and anxiety, and facilitate children's expression of preferences and concerns.

This article also emphasizes the importance of the nondiscrimination principle in Article 2 of the UNCRC for realizing the right to play under Article 31. Article 2 obliges States Parties, including Chile, to respect and protect children's rights without discrimination based on social background, disability, or health status. From a rights-based view, the limited incorporation of play into public healthcare policy and practice could raise concerns about indirect discrimination, especially when such restrictions disproportionately impact children who are disabled, chronically ill, or hospitalized for long periods. Scholarship highlights that play is vital to children's wellbeing, development, and dignity (19, 94–96). Studies also show that limited access to play can disadvantage children's development compared to their peers (20, 97, 98). From a practical perspective, this requires paying particular attention to children who experience prolonged hospitalization, isolation, or limited mobility, for whom access to play may be especially restricted. Hospitals should ensure that play opportunities are accessible to children with diverse physical, cognitive, sensory, and emotional needs through inclusive design, adapted materials, and appropriately trained staff. Play should not become another site of exclusion or inequality within healthcare systems. Rather, it can serve as an inclusive practice that supports children's flourishing, participation, and sense of belonging across diverse age, gender, social background, health condition, or abilities, as such contributing to the creation of a more just society, also beyond hospital walls.

## Data Availability

The raw data supporting the conclusions of this article will be made available by the authors, without undue reservation.

## Footnotes

^1^In this community, municipal estimates indicate an income poverty rate of 1.3%, and the multidimensional poverty rate, which assesses deprivation across various aspects like education, health, and living conditions, of 4.3% (99).

^2^Within this community, municipal estimates show an income poverty rate of 5.0% and a multidimensional poverty rate of 18.9% (Casen, 2022).

^3^In Araucanía, recent data from the Socioeconomic Characterization Survey (CASEN, 2024) shows a total poverty rate of 28.6%, which includes 13% experiencing extreme poverty and 15.5% facing non-extreme poverty. Additionally, multidimensional poverty impacts 15.9% of the population, with higher rates in rural areas, among Indigenous peoples, women, and children. The 2024 Census (101) reports that 347,285 people in Araucanía identify as Indigenous or Native American, representing 34% of the population. Of these, 344,445 identify as Mapuche (99%), and 74,930 are under 15 years old.

^4^The region also faces considerable socioeconomic challenges: 21.4% of the population lives in poverty, including 9.9% in extreme poverty, while multidimensional poverty affects 18.7% of the population (CASEN, 2024).
