# Supplementary material for: Child-centred care in practice: strengthening the right to play in public hospitals in Chile
Source: Front Health Serv. 2026 Jun 22;6:1824874. doi: 10.3389/frhs.2026.1824874 (PMC13348202; doi:10.3389/frhs.2026.1824874)
Supplement: Supplementary file 1 [file Table1.docx]

**Braun & Clarke 15-point Thematic Analysis Checklist**

| **Process** | **No.** | **Criteria** | **Response** |
| --- | --- | --- | --- |
| **Transcription** | 1 | The data have been transcribed to an appropriate level of detail, and the transcripts have been checked against the tapes for ‘accuracy’ | All interviews were transcribed verbatim by a professional transcriber under a confidentiality agreement. The transcripts were subsequently checked against the audio recordings and corrected by the first author to ensure accuracy. |
| **Coding** | 2 | Each data item has been given equal attention in the coding process | All interviews were carefully read, re-read, and analysed to develop codes, ensuring equal attention across the dataset. |
|  | 3 | Themes have not been generated from a few vivid examples (an anecdotal approach), but instead the coding process has been thorough, inclusive and comprehensive | Themes were developed through thorough and comprehensive coding of the entire dataset, with all participant quotations considered. Each theme was constructed from multiple codes drawn across interviews with participants from different hospitals. |
|  | 4 | All relevant extracts for each theme have been collated | Five analytical matrices (one for each hospital and, separately, for ethnographic data) were developed from the full dataset, collating all relevant extracts for each theme. This matrix is available in the data repository. |
|  | 5 | Themes have been checked against each other and back to the original data set | Yes, in a reiterative and collaborative process by the research team. |
|  | 6 | Themes are internally coherent, consistent, and distinctive | The themes are coherent, conceptually distinct, and interdependent, mutually informing one another while each contributes a unique aspect to the phenomenon under study. |
| **Analysis** | 7 | Data have been analysed- interpreted, made sense of- rather than just paraphrased or described | Yes, evident from the results. |
|  | 8 | Analysis and data match each other- the extracts illustrate the analytic claims | Yes, our findings are strongly supported by the evidence |
|  | 9 | Analysis tells a convincing and well- organised story about the data and topic | Yes, evident from the results. |
|  | 10 | A good balance between analytical narrative and illustrative extracts is provided | Analytical narrative and illustrative data extracts are presented in a balanced, creative manner throughout the text, tables, and photographs. |
| **Overall** | 11 | Enough time has been allocated to complete all phases of the analysis adequately, without rushing a phase or giving it a once-over-lightly | Yes. The analysis was a thorough, iterative process conducted over six months with multiple review and refinement rounds. Regular meetings and written feedback on analytic matrices supported collaborative interpretation, ensuring rigour and responsible use of trust placed in the research team by participating health professionals. |
| **Written report** | 12 | The assumptions about, and specific approach to, thematic analysis are clearly explicated | Stated in the methods section. |
|  | 13 | There is a good fit between what you claim you do, and what you show you have done- i.e. described method and reported analysis are consistent | Yes, as evident from the methods and results. |
|  | 14 | The language and concepts used in the report are consistent with the epistemological position of the analysis | We adopt an interpretive rather than a strictly positivist language style. |
|  | 15 | The researcher is positioned as active in the research process; themes do not just ‘emerge’ | Yes, this methodical approach was chosen because it supports an interpretive, while theoretically informed analysis, while recognising researchers’ active engagement with data and analysis. |

REFERENCE:

1. Braun V, Clarke V. Successful qualitative research: a practical guide for beginners. London: SAGE Publications Ltd; 2013, p. 287.
