# Supplementary material for: Child-centred care in practice: strengthening the right to play in public hospitals in Chile
Source: Front Health Serv. 2026 Jun 22;6:1824874. doi: 10.3389/frhs.2026.1824874 (PMC13348202; doi:10.3389/frhs.2026.1824874)
Supplement: Supplementary file 3 [file Table3.docx]

Standard 3: Play and Learning

|  | **(Convention on the Rights of the Child, Articles 23, 28, 29 and 31)** | | | | |  |  |
| --- | --- | --- | --- | --- | --- | --- | --- |
|  |  |  | **Elements to assess** | **H1** | **H2** | **H3** | **H4** |
|  |  | **Significant progress** | 1. Evidence shows that children of all ages have opportunities to play and leisure in accordance to their age and preferences (i.e. both younger children and adolescents). | 100% | 50% | 78% | 100% |
|  |  |  | 2. The hospital provides other supportive activities such as clown, music, art and/or pet-therapy or similar. | 100% | 100% | 88% | 67% |
|  |  |  | 3. All doctors and nurses utilise play within therapeutic care. | 0% | 0% | 0% | 0% |
|  |  |  | 4. Children’s views were collected during the planning of the  playroom or they have been consulted at a later stage about the appropriateness of the space and how to improve it. | 50% | 75% | 13% | 33% |
| **Progressive implementation of children’s right to play and learning** | |  | 5. Evidence gathered from children and parents show that they are satisfied with the available play services. | 25% | 50% | 50% | 0% |
|  |  |  | 6. The hospital promotes research about the benefits of using play during therapeutic care or other supportive activities promoted, which are published and shared with wider  audiences. | 60% | 25% | 44% | 0% |
|  |  | **Meaningful progress** | 7. There is a hospital policy guaranteeing children’s right to play. | 80% | 75% | 22% | 50% |
|  |  |  | 8. There is a properly equipped playroom. | 20% | 25% | 11% | 100% |
|  |  |  | 9. There are play specialists to support children during play. | 80% | 50% | 44% | 67% |
|  |  |  | 10. Every child is encouraged and helped to play, even if they cannot leave their bed. | 75% | 25% | 33% | 67% |
|  |  |  | 11. Most doctors and nurses have received training on how to utilise play within therapeutic care and they apply it. | 0% | 13% | 0% | 0% |
|  |  |  | 12. There is a hospital school, trained teacher or another system enabling children to continue their education whilst in hospital. | 100% | 100% | 100% | 100% |
|  |  | **Some action** | 13. A play policy is under development. | 75% | 43% | 33% | 0% |
|  |  |  | 14. There is no playroom for children, but there is a space where children can go and play with other children. | 60% | 88% | 33% | 0% |
|  |  |  | 15. Play is utilised within therapeutic care by some  professionals that have undertaken related training. | 100% | 75% | 56% | 50% |
|  |  |  | 16. There are some possibilities for children to continue their education whilst in hospital. | 100% | 100% | 100% | 100% |
|  |  | **No action** | 17. There are no internal policies guaranteeing children’s right to play. | 0% | 50% | 78% | 0% |
|  |  |  | 18. There is no playroom for children. | 60% | 38% | 78% | 50% |
|  |  |  | 19. There are no specialised play staff in the hospital (i.e. play specialists). | 40% | 50% | 100% | 0% |
|  |  |  | 20. Play is not utilised within therapeutic care (i.e. to stimulate development, in preparation for procedures, distraction or  helping a child to express their feelings). | 20% | 13% | 33% | 50% |
|  |  |  | 21. There is no possibility for children to continue their  education whilst in hospital (i.e. through a hospital school, a trained teacher or another enabling system). | 20% | 50% | 11% | 100% |
